# Role of hypothermia in the immediate postoperative period on mortality in a surgical ICU

**DOI:** 10.1186/cc12675

**Published:** 2013-06-19

**Authors:** AR Santana, FF Amorim, FB Soares, LG de Souza Godoy, L de Jesus Almeida, TA Rodrigues, GM de Andrade Filho, TA Silva, LC de Carvalho Santos, MPB de Araújo, PN Ferreira, APP Amorim, EB de Moura, JA de Araújo Neto, M de Oliveira Maia

**Affiliations:** 1 Unidade de Terapia Intensiva Adulto do Hospital Santa Luzia, Asa Sul, Brasília, DF, Brazil

## Introduction

Surgical patients are submitted to numerous factors that may cause postoperative hypothermia, such as a cool operating room environment, cold intravenous fluids and blood, cold antiseptic skin preparations and anesthetic-induced impairment of thermoregulatory control. Hypothermia may increase susceptibility to surgical wound infection, length of stay, intraoperative blood loss, morbid cardiac events, postoperative shivering, coagulopathy and also altered duration of drug action. The objective of this study was to evaluate the impact of hypothermia at ICU admission on hospital length of stay and mortality in a surgical ICU.

## Methods

A prospective cohort study conducted on patients admitted to the ICU of Hospital Santa Luzia, Brasilia, Brazil, during the period of 1 year. Hypothermia was defined as axillary temperature inferior to 35.5°C (95.9°F). Patients were divided into groups with hypothermia (HG) and without hypothermia (NG).

## Results

A total of 484 patients were enrolled. Mean age was 59 ± 16 years and 52.5% were male. Seventy-eight patients (16.1%) were submitted to emergency surgeries. Mean APACHE II score was 8 ± 5, mean SAPS II was 16 ± 10. Twenty-four patients (5%) had hypothermia at the time of ICU admission. The general mortality rate at 7 days, 28 days and hospital mortality was 0.8% (*n = *4), 1.9% (*n = *9) and 4.1% (*n = *20), respectively. Patients with hypothermia had higher APACHE II score (15 ± 12 vs. 8 ± 4, *P *= 0.00) and SAPS II (25 ± 17 vs. 26 ± 10, *P *= 0.00). There was no difference regarding the age of the groups and the hospital length of stay (4 ± 6 days in NG vs. 5 ± 12 days in HG, *P *= 0.76). However, the group with hypothermia had higher mortality rates (20% vs. 4.3%, *P *= 0.00). The relative risk for hospital mortality in patients with hypothermia at ICU admission was 4.6 (95% CI: 1.02 to 29.88).

## Conclusion

Few patients were hypothermic at the time of ICU admission in the immediate postoperative period. This may reflect the effectiveness of perioperative warming of the patients. Hypothermia at ICU admission was associated with greater severity scores and increased hospital mortality in this sample of surgical patients studied.
